# Pyruvate Carboxylase Activates the RIG-I-like Receptor-Mediated Antiviral Immune Response by Targeting the MAVS signalosome

**DOI:** 10.1038/srep22002

**Published:** 2016-02-24

**Authors:** Zhongying Cao, Yaqin Zhou, Shengli Zhu, Jian Feng, Xueyuan Chen, Shi Liu, Nanfang Peng, Xiaodan Yang, Gang Xu, Ying Zhu

**Affiliations:** 1 State Key Laboratory of Virology and College of Life Sciences, Wuhan University, Wuhan 430072, China

## Abstract

When retinoic acid-inducible gene 1 protein (RIG-I)-like receptors sense viral dsRNA in the cytosol, RIG-I and melanoma differentiation-associated gene 5 (MDA5) are recruited to the mitochondria to interact with mitochondrial antiviral signaling protein (MAVS) and initiate antiviral immune responses. In this study, we demonstrate that the biotin-containing enzyme pyruvate carboxylase (PC) plays an essential role in the virus-triggered activation of nuclear factor kappa B (NF-κB) signaling mediated by MAVS. PC contributes to the enhanced production of type I interferons (IFNs) and pro-inflammatory cytokines, and PC knockdown inhibits the virus-triggered innate immune response. In addition, PC shows extensive antiviral activity against RNA viruses, including influenza A virus (IAV), human enterovirus 71 (EV71), and vesicular stomatitis virus (VSV). Furthermore, PC mediates antiviral action by targeting the MAVS signalosome and induces IFNs and pro-inflammatory cytokines by promoting phosphorylation of NF-κB inhibitor-α (IκBα) and the IκB kinase (IKK) complex, as well as NF-κB nuclear translocation, which leads to activation of interferon-stimulated genes (ISGs), including double-stranded RNA-dependent protein kinase (PKR) and myxovirus resistance protein 1 (Mx1). Our findings suggest that PC is an important player in host antiviral signaling.

The innate immune response is a critical host defense system against microbial pathogen invasion. Following infection, bacteria and viruses are initially sensed by the pattern-recognition receptors (PRRs), which include Toll-like receptors (TLRs) and RIG-I-like helicases^1^,^2^,^3^. TLRs are type I transmembrane proteins with ectodomains containing leucine-rich repeats, which mediate the recognition of pathogen-associated molecular patterns; a transmembrane region; and cytosolic Toll-interleukin-1 receptor (TIR) domains that activate downstream signaling pathways^4^,^5^. The RLR signaling pathway is initiated by the recognition of distinct viral RNA species by one of two cytosolic sensors RIG-I (also known as DDX58) or MDA5 (also known as IFIH1)^6^. RIG-I, which is the first identified sensor of the RLR pathway, is composed of several domains, including two amino-terminal (N-terminal) caspase activation and recruitment domains (CARDs), a central DEAD box helicase/ATPase domain, and a C-terminal regulatory domain^7^,^8^. MDA5 shares structural homology with RIG-I in that it contains two N-terminal CARD domains and a central DEAD box helicase/ATPase domain^9^,^10^. Viral RNA binding enables the N-terminal-CARDs of RLRs to interact with the adaptor molecule MAVS (alternatively known as IPS-1/CARDIF/VISA), a mitochondrial outer membrane protein composed of a N-terminal single CARD, a central proline-rich region that contains two tumor necrosis factor receptor-associated factor (TRAF) binding motifs and a transmembrane domain (TM)^3^,^11^,^12^,^13^,^14^. MAVS plays an essential role in RIG-I signaling pathway. Many studies indicate how MAVS regulates the innate immune response. MAVS recruits several adaptors to assemble a MAVS “signalosome” including TRAF3, TRAF6, TRAF family member-associated NF-κB activator (TANK), and TNFR1-associated death domain protein (TRADD)^15^,^16^,^17^, which activates the interferon regulatory factor (IRF) 3/7 and NF-κB^2^,^3^, and eventually leads to the production of IFNs and pro-inflammatory cytokines^1^,^18^,^19^. Recently, it is reported that the initial ubiquitination and subsequent phosphorylation on MAVS are necessary for IRF3 interaction and IFNs induction^20^. Insulin receptor tyrosine kinase substrate negatively modulates the excessive inflammation response through mediates MAVS degradation^21^. Moreover, secreted type I IFNs bind to cognate receptors on the surface of surrounding cells to activate the JAK/STAT pathway and transcriptional induction of a wide range of ISGs. The induced downstream gene products such as PKR, Mx1, and OAS, orchestrate the inhibition of viral replication and clearance of virus-infected cells that lead to antiviral responses^22^,^23^,^24^.

Pyruvate carboxylase (PC) is a member of biotin-containing enzyme family that catalyzes the ATP-dependent carboxylation of pyruvate to oxaloacetate^25^. This is a very important anaplerotic reaction for various pivotal biochemical pathways in the central metabolism. Therefore, this reaction plays an important role in numerous biological processes, including de novo fatty acid synthesis in liver and adipose tissue, glyceroneogenesis in adipose tissue, and glutamate production in astrocytes^26^,^27^,^28^. In addition, a recent study indicated that PC-mediated anaplerosis is required for tumor survival and proliferation in early-stage non-small-cell lung cancer^29^. The distal promoter of the human PC gene has also been described^30^. PC deficiency is a rare autosomal recessive phenotype characterized by mild to severe lactic acidemia associated with delayed psychomotor development and death within the first year of life in about half the cases^31^. PC has been found in a wide variety of prokaryotes and eukaryotes and exists in two forms, α4 and α4β4, depending on the organism. The α4β4 type is found in archaebacteria and some bacteria, while the α4 type is found in most organisms ranging from eubacteria and fungi to invertebrates and vertebrates. The α4 form is comprised of four identical subunits, each approximately 120–130 kDa^26^. The three functional domains, biotin carboxylase (BC), carboxyltransferase (CT), and biotin carboxyl carrier protein (BCCP), are located on a single polypeptide chain^25^. The BC domain is at the N-terminus of the polypeptide chain and is responsible for binding biotin to carboxylate bicarbonate. The CT domain catalyzes the transfer of a carboxyl group from carboxybiotin to the accepting substrate, pyruvate. The BCCP domain at the C-terminus contains the lysine residue, to which biotin is attached^26^.

Although multiple functions of PC have been clearly described, its role in innate immunity against viral infection has never been established. In this study, we demonstrate that PC represses the replication of RNA viruses such as influenza A virus (IAV), vesicular stomatitis virus (VSV), and human enterovirus 71 (EV71) by targeting the MAVS signalosome to activate NF-κB signaling. Our results describe a previously unrecognized function of PC.

## Results

### PC potentiates virus-triggered interferon induction

**Figure 1:**
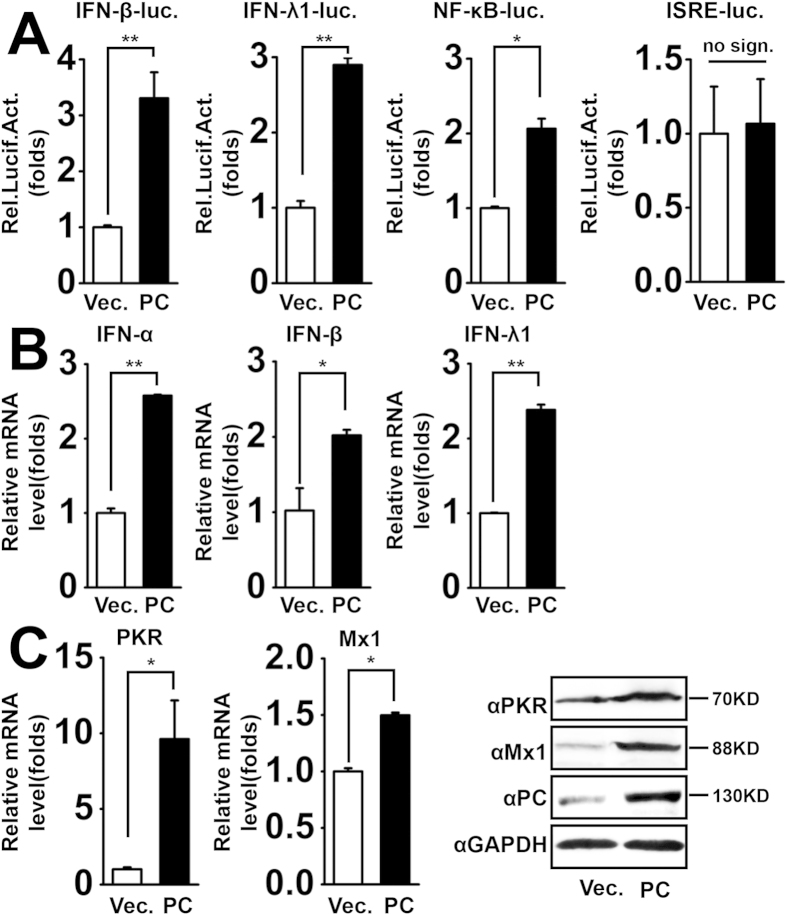
PC potentiates virus-triggered interferon induction. (**A**) A549 cells were co-transfected with IFN-β, IFN-λ1, NF-κB, and ISRE luciferase reporter constructs and control vector or PC expression plasmids. pRL-TK was cotransfected as an internal control in the luciferase assay. Twenty-four hours after transfection, cells were infected with SeV (MOI = 1) for 12 h before reporter assays were performed. *p < 0.05, **p < 0.01. no sign., no significant difference (one-way ANOVA). (**B**) A549 cells were transfected with vector or PC expression plasmids for 24 h and then infected with SeV (MOI = 1) for 6 h. IFN mRNA levels were measured by qPCR. *p < 0.05, **p < 0.01 (one-way ANOVA). (**C**) A549 cells were transfected with the vector or PC expression plasmids for 24 h and then infected with SeV (MOI = 1) for 12 h. PKR and Mx1 mRNA levels were determined by qPCR. PKR, Mx1 and PC proteins were assessed by western blot, respectively. *p < 0.05 (one-way ANOVA). All graphs represent means standard deviations for 3 experiments.

To explore the potential role of PC proteins in regulating IFN expression, we performed reporter assays in A549 cells infected with SeV (MOI = 1). In reporter assays, PC overexpression potentiated virus-induced activation of the IFN-β and IFN-λ1 promoters in A549 cells (Fig. 1A). PC expression induced NF-κB reporter luciferase activity but not that of ISRE (Fig. 1A), indicating that PC is only involved in activating NF-κB signaling. Next, we detected mRNA expression of different types of IFN. The qPCR results indicated that PC overexpression up-regulated virus-induced mRNA levels of IFN-α, IFN-β, and IFN-λ1 (Fig. 1B). We also assessed the IFN downstream effectors (the ISGs, including PKR and Mx1), by qPCR and western blot. The results showed that PC overexpression increased the mRNA and protein levels of PKR and Mx1 (Fig. 1C). Collectively, these data suggest that PC positively regulates virus-induced IFN production and IFN downstream effectors.

### PC is required for virus-triggered expression of IFNs and ISGs

**Figure 2:**
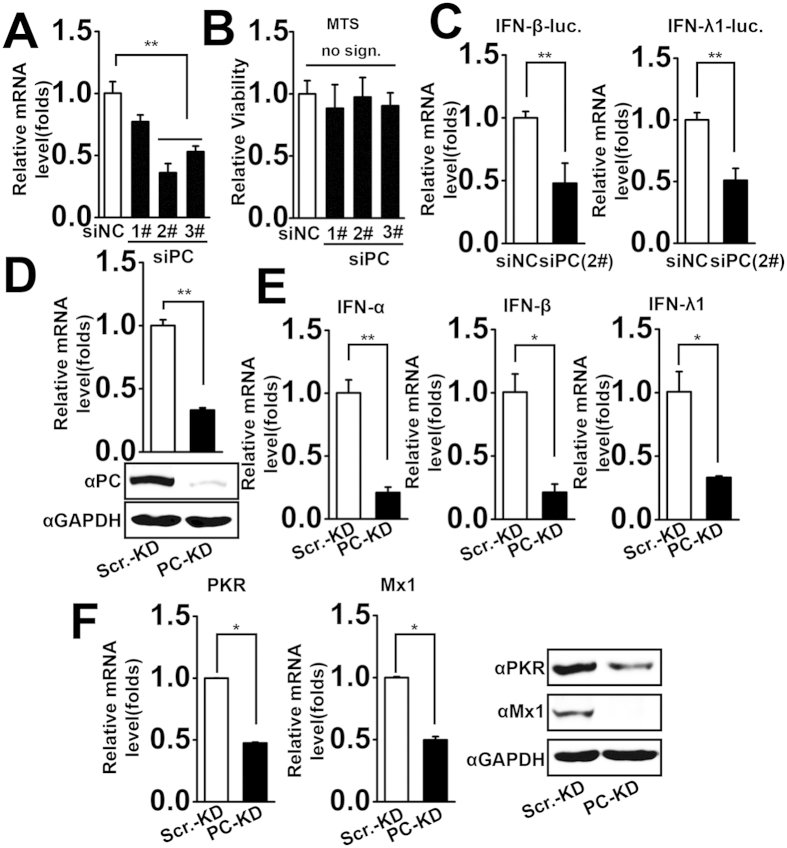
PC is required for virus-triggered expression of IFN and ISG. (**A**) A549 cells were transfected with PC-specific siRNA or nonsense control siRNA for 48 h, and PC mRNA levels were determined by qPCR. **p < 0.01 (one-way ANOVA). (**B**) A549 cells were transfected with different PC-specific siRNA and control siRNA. Fourty-eight hours after transfection, MTS assays were performed using a MTS cell viability kit (Promega) according to the manufacturer’s instructions. Cell viability was then calculated. no sign., no significant difference (one-way ANOVA). (**C**) A549 cells were co-transfected with the indicated PC-specific siRNA or nonsense control siRNA and IFN-β or IFN-λ1 luciferase reporter plasmids for 24 h. pRL-TK was cotransfected as an internal control in the luciferase assay. Luciferase assays were performed after cells were infected with SeV (MOI = 1) for 12 h. **p < 0.01 (one-way ANOVA). (**D**) PC-KD and Scr.-KD cells were collected to assess PC mRNA and protein levels by qPCR and western blot, respectively. **p < 0.01 (one-way ANOVA). (**E**) The same numbers of PC-KD and Scr.-KD cells were infected with SeV (MOI = 1) for 6 h. IFN mRNA levels were measured by qPCR. *p < 0.05, **p < 0.01 (one-way ANOVA). (**F**) PC-KD and Scr.-KD cells were infected with SeV (MOI = 1) for 12 h. PKR and Mx1 mRNA and protein levels were determined by qPCR and western blot, respectively. *p < 0.05 (one-way ANOVA). All graphs represent means standard deviations for 3 experiments.

Because PC overexpression potentiates virus-triggered IFN induction, we further determined whether endogenous PC is required for this process. Three specific siRNAs for PC (siPC Nos. 1#-3#) were designed, and siPC-2# and siPC-3# of them effectively knocked down PC mRNA (Fig. 2A). The transfection of all PC-specific-siRNAs had little influence on cell viability of A549 cells (Fig. 2B). As the siPC-2# has the best efficiency on the knockdown of PC expression, we chose siPC-2# to perform the follow-up experiments. Results from the reporter assays indicated that PC knockdown suppressed virus-triggered activation of IFN-β and IFN-λ1 promoter activity (Fig. 2C). To further confirm the role of endogenous PC in regulating IFN expression, we generated a stable PC-silenced A549 cell line using a vector-based shRNA in a lentiviral system^32^. PC was significantly reduced at both mRNA and protein levels in cells transduced with recombinant lentivirus containing specific PC-shRNA (PC-KD) compared to those infected with recombinant lentivirus containing scramble shRNA (Scr.-KD) (Fig. 2D). Consistent with the siRNA experiments, PC-KD cells showed lower IFN-α, IFN-β, and IFN-λ1 mRNA levels than those in Scr.-KD cells upon virus infection (Fig. 2E). Subsequently, we assessed the effect of PC depletion on virus-induced expression of ISGs, including PKR and Mx1, by qPCR and western blot analyses in PC-KD cells. The results indicated that PC depletion decreased both mRNA and protein levels of PKR and Mx1 (Fig. 2F). Taken together, these data suggest that endogenous PC plays an essential role in virus-triggered IFN and ISG expression.

### Identification of PC as a positive regulator of virus-induced pro-inflammatory cytokines

**Figure 3:**
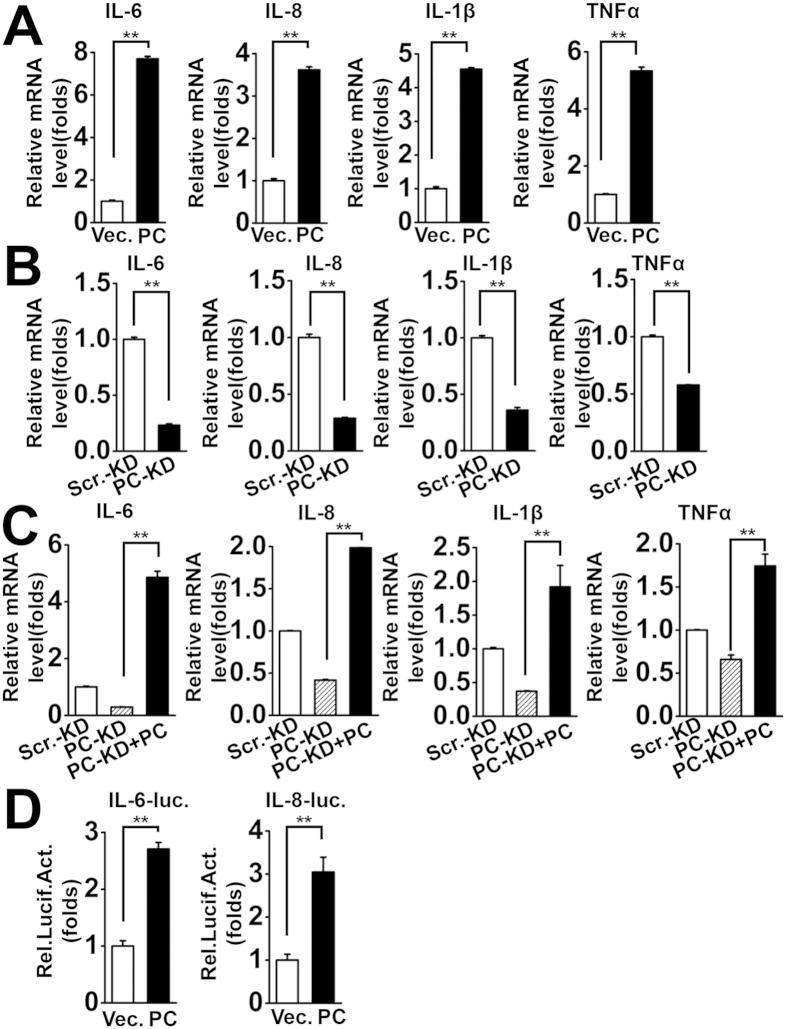
Identification of PC as a positive regulator of virus-induced pro-inflammatory cytokines. (**A**) A549 cells were transfected with vector or PC expression plasmids for 24 h and then infected with SeV (MOI = 1) for 6 h. IL-6, IL-8, IL-1β, and TNFα mRNA levels were measured by qPCR. (**B**) The same numbers of PC-KD and Scr.-KD cells were infected with SeV (MOI = 1) for 6 h. IL-6, IL-8, IL-1β, and TNFα mRNA levels were measured by qPCR. (**C**) PC-KD and Scr.-KD cells were transfected with vector or PC expression plasmids for 24 h and then infected with SeV (MOI = 1) for 6 h. IL-6, IL-8, IL-1β, and TNFα mRNA levels were measured by qPCR. (**D**) A549 cells were co-transfected with IL-6 or IL-8 luciferase reporter plasmids, and vector or PC expression plasmids. pRL-TK was cotransfected as an internal control in the luciferase assay. Twenty-four hours after transfection, cells were infected with SeV (MOI = 1) for 12 h before reporter assays were performed. **p < 0.01 (one-way ANOVA). All graphs represent means standard deviations for 3 experiments.

Since we found that PC potentiates virus-triggered activation of the NF-κB promoter, we speculated that PC up-regulates the production of NF-κB-responsive genes encoding pro-inflammatory cytokines, including IL-6, IL-8, IL-1β, and TNFα. To test this hypothesis, we investigated the effect of PC on pro-inflammatory cytokines expression in A549 cells upon virus infection. The results indicated that PC overexpression enhanced virus-induced production of pro-inflammatory cytokines (IL-6, IL-8, IL-1β, and TNFα) at the mRNA level (Fig. 3A). To further investigate the potential role of endogenous PC in this process, we detected the expression of IL-6, IL-8, IL-1β, and TNFα in PC-KD cells infected with SeV (MOI = 1). The results showed that PC depletion significantly decreased mRNA levels of IL-6, IL-8, IL-1β, and TNFα (Fig. 3B). To confirm that PC depletion in PC-KD cells was responsible for the reduced expression of pro-inflammatory cytokines, we transfected PC expression plasmids into PC-KD cells to restore PC production. Interestingly, restoration of PC in PC-KD cells rescued the reduced productions of IL-6, IL-8, IL-1β, and TNFα (Fig. 3C). The reporter assays also indicated that PC enhanced the virus-triggered activation of the IL-6 and IL-8 promoters (Fig. 3D). Because PC is a biotin-containing enzyme that catalyzes the ATP-dependent carboxylation of pyruvate to oxaloacetate, and participates in the central metabolism, we then test whether the enzymatic activity of PC is required for its activation of NF-κB. PC inhibitor phenylacetic acid (PAA) was used to inhibit influx of glucose and free fatty acids into the tricarboxylic acid (TCA) cycle^33^,^34^,^35^. The PC activity assays indicated that PAA inhibited the PC activity effectively (Supplemental Fig. 1A). However, PAA treatment had no significant effect on PC-activated expression of IL-6 and IL-8 (Supplemental Fig. 1B). Taken together, these data show that PC up-regulates virus-triggered activation of pro-inflammatory cytokines.

### PC positively regulates the host antiviral response

**Figure 4:**
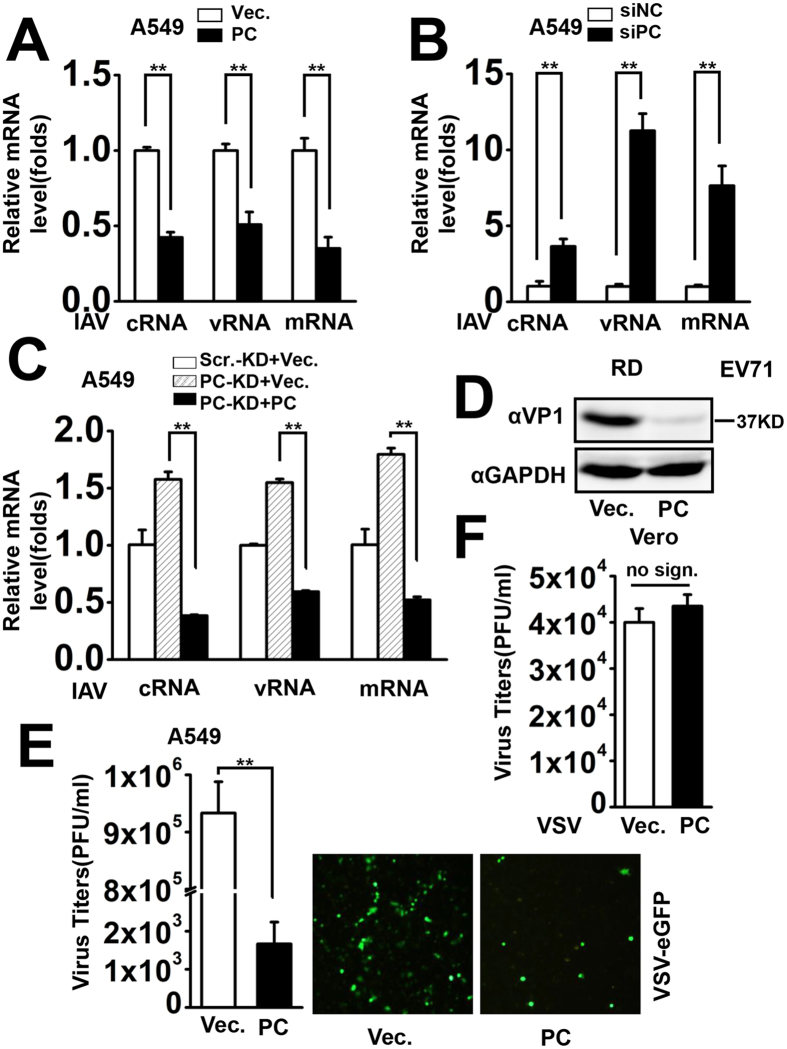
PC positively regulates cellular antiviral response. A549 cells were transfected with vector or PC expression plasmids for 24 h were infected with IAV (MOI = 1) for another 24 h, and cells were harvested for total RNA isolation. Relative levels of NP-specific mRNA, cRNA, and vRNA were measured by qPCR. **p < 0.01 (one-way ANOVA). (**B**) A549 cells were transfected with PC-specific siRNA or nonsense control siRNA for 24 h. Cells were harvested 24 h after infection with IAV (MOI = 1). Relative levels of NP-specific mRNA, cRNA, and vRNA were measured by qPCR. **p < 0.01 (one-way ANOVA). (**C**) PC-KD and Scr.-KD cells were transfected with vector or PC expression plasmids for 24 h. The cells were harvested 24 h after IAV infection (MOI = 1), and the relative levels of NP-specific mRNA, cRNA, and vRNA were measured by qPCR. **p < 0.01 (one-way ANOVA). (**D**) RD cells were transfected with vector or PC expression plasmids for 24 h. Twelve hours after EV71 infection (MOI = 1), VP1 protein levels were determined by western blot. (**E**) A549 cells were transfected with vector or PC expression plasmids for 24 h and then infected with VSV-eGFP (MOI = 1) for 6 h. VSV-eGFP replication was visualized by fluorescence microscopy (right panel). Twenty-four hours post-infection, the supernatants were harvested and analyzed for VSV production using a standard plaque assay (left panel). **p < 0.01. (**F**) Vero cells were transfected with vector or PC expression plasmids for 24 h and then infected with VSV (MOI = 1). VSV production in the supernatants was estimated as in (**E**) no sign., no significant difference. All graphs represent means standard deviations for 3 experiments.

Because our results demonstrated that PC plays a key role in virus-triggered IFN and pro-inflammatory cytokine expression, we wondered whether it is involved in the host antiviral action. Different virus infection systems including IAV, EV71 and VSV were applied to test the antiviral activity of PC. The results showed that PC expression significantly suppressed IAV NP gene levels, including cRNA, vRNA, and mRNA, which were all measured by qPCR (Fig. 4A). Conversely, viral replication was significantly promoted by specific PC siRNA (Fig. 4B). To further confirm the role of PC, we examined IAV replication in PC-KD cells. Consistent with the siRNA results, IAV displayed enhanced replication in PC-KD cells compared to Scr.-KD cells. However, viral replication was significantly inhibited when PC was restored in PC-KD cells by transfection of the PC expression plasmid (Fig. 4C).

Viral VP1, a capsid protein of EV71, was detected by western blot, and the results showed that PC expression evidently inhibited EV71 expression in RD cells (Fig. 4D). Subsequently, we examined the effects of PC on the production of the recombinant virus VSV-eGFP in A549 cells. Viral titers were significantly lower in PC-overexpressing cells compared to control cells (Fig. 4E, left). Moreover, VSV-eGFP replication was visualized by fluorescence microscopy, and it was obvious that PC expression inhibited VSV-eGFP expression (Fig. 4E, right). Thus, we showed that PC has a universal antiviral function against RNA viruses (IAV, EV71, and VSV). In addition, VSV was infected into Vero cells, a cell line lacking functional type I IFN genes^36^,^37^, to examine the effect of PC on viral replication. The results indicated that VSV replication was not affected in Vero cells, implying that PC exerted its antiviral function through the IFN signaling pathway (Fig. 4F).

### PC regulates virus-triggered signaling through MAVS

**Figure 5:**
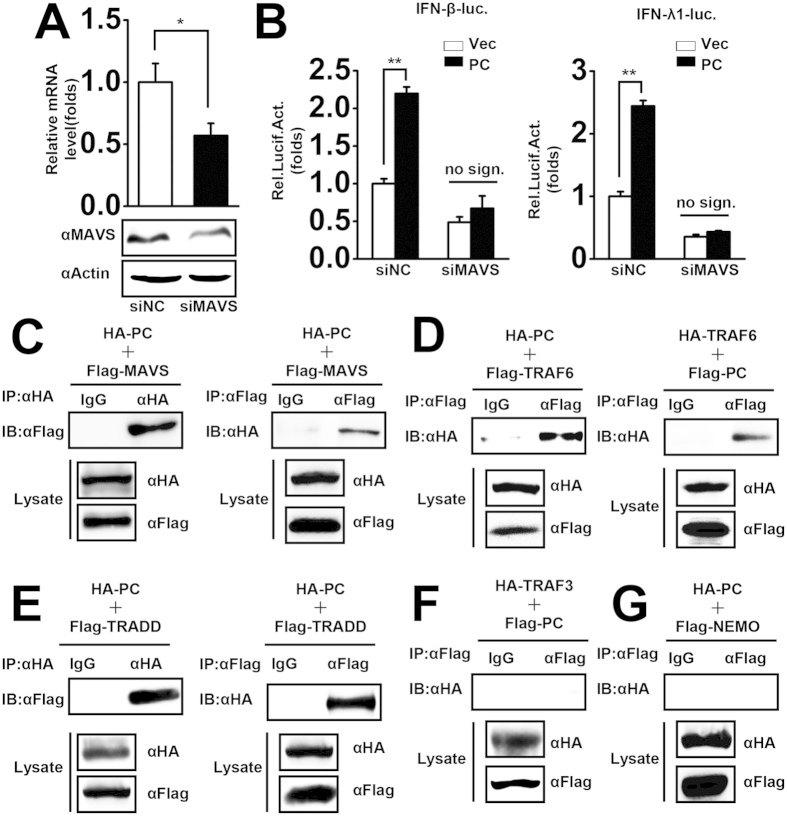
PC regulates virus-triggered signaling through MAVS. (**A**) A549 cells were transfected with MAVS-specific siRNA or nonsense control siRNA for 48 h, and MAVS mRNA and protein levels were determined by qPCR and western blotting, respectively. *p < 0.05 (one-way ANOVA). (**B**) A549 cells were co-transfected with vector or PC expression plasmids and the indicated MAVS-specific siRNA or nonsense control siRNA together with IFN-β and IFN-λ1 luciferase reporter plasmids. pRL-TK was cotransfected as an internal control in the luciferase assay. Twenty-four hours after transfection, cells were infected with SeV (MOI = 1) for 12 h before reporter assays were performed. *p < 0.05, **p < 0.01 (one-way ANOVA). HEK293T cells were co-transfected with HA-PC and Flag-MAVS expression plasmids (**C**), HA-TRAF6 and Flag-PC or Flag-TRAF6 and HA-PC expression plasmids (**D**), HA-PC and Flag-TRADD expression plasmids (**E**), HA-TRAF3 and Flag-PC expression plasmids (**F**), or HA-PC and Flag-NEMO expression plasmids (**G**) for 48 h. Co-immunoprecipitation and immunoblot analyses were performed with the indicated antibodies. All experiments were repeated at least three times with consistent results.

MAVS, located on mitochondria, is one of the key proteins in the RLR signaling pathway, through which the host senses and responds to invading pathogens, such as IAV, VSV, and SeV^14^,^38^,^39^. Since our results indicate that PC potentiates RNA virus-triggered IFNs and pro-inflammatory cytokines expression, then we asked whether MAVS was required for PC activated IFNs. MAVS-specific siRNA was synthesized and the knockdown efficiency was tested at both mRNA and protein levels (Fig. 5A). In report assay, the results showed that the knockdown of MAVS significantly inhibited PC-induced IFNβ and IFNλ1 promoter activities (Fig. 5B), implying that PC mediated increase of IFNs expression upon virus infection is MAVS-dependent. Furthermore, we investigated the crosstalk between PC and MAVS-associated components in virus-triggered signaling pathways. Specifically, in transient transfection and co-immunoprecipitation experiments investigate the interaction between PC and MAVS-associated components. The results indicated that HA-tagged PC interacted with Flag-tagged MAVS. Reciprocal co-immunoprecipitation results also indicated that Flag-tagged MAVS interacted with HA-tagged PC (Fig. 5C). Furthermore, HA-tagged TRAF6 interacted with Flag-tagged PC (Fig. 5D), and HA-tagged PC interacted with Flag-tagged TRADD (Fig. 5E). PC did not interact with TRAF3 (Fig. 5F) or NEMO (Fig. 5G), which were other two MAVS-associated components. These data suggest that PC targets the MAVS-TRAF6 signaling pathway, which is consistent with our finding that PC activates the NF-κB reporter but not ISRE.

### Virus triggers PC cytoplasmic translocation and interacts with MAVS and TRAF6

**Figure 6:**
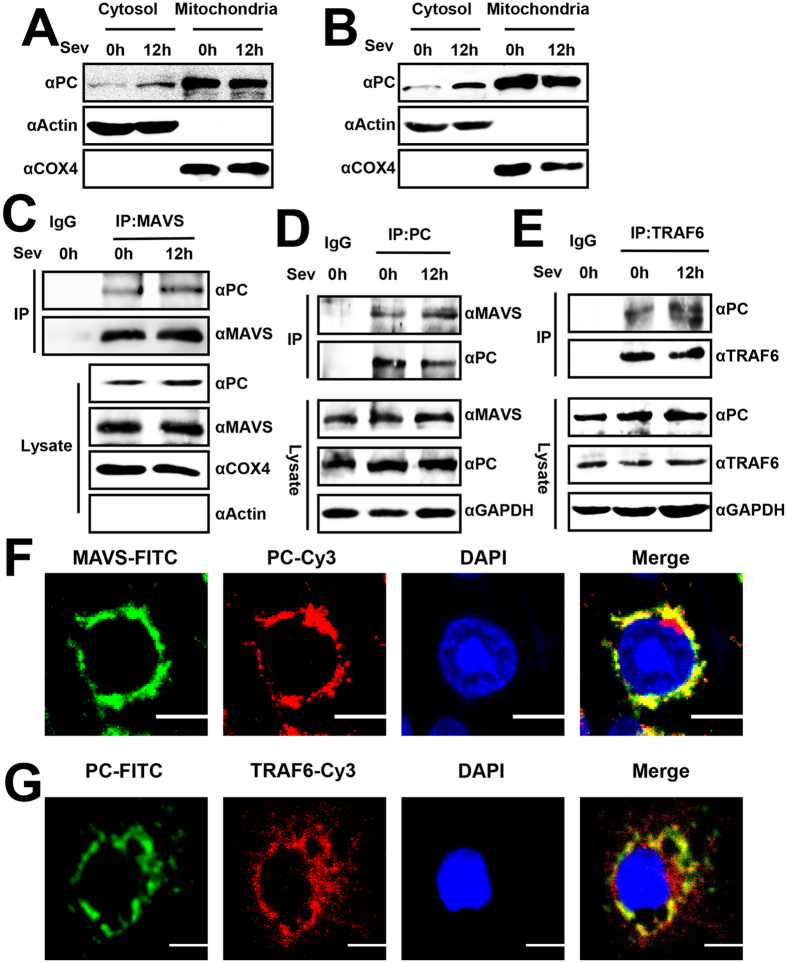
Virus triggers PC cytoplasmic translocation and PC interacts with MAVS and TRAF6. (**A**) A549 cells were infected with SeV (MOI = 1) for 12 h or left uninfected. Mitochondrial extractions were performed and the fractions were analyzed by western blotting with the indicated antibodies. (**B**) 293T cells were infected with SeV (MOI = 1) for12 h or left uninfected. Western blotting was performed as in (**A**). (**C**) 293T cells were infected with SeV (MOI = 1) for12 h or left uninfected. Isolation of Mitochondria was then performed and the mitochondrial extracts were prepared by lysing mitochondrion in RIPA buffer. Co-immunoprecipitation and immunoblot analyses were performed with the MAVS, PC, Actin or COX4 antibodies. (**D**) A549 cells were transfected with PC or vector plasmids for 24 h. The cells were harvested after they were infected with SeV (MOI = 1) or left uninfected for 12 h. Co-immunoprecipitation and immunoblot analyses were performed with the MAVS, PC or GAPDH antibodies. (**E**) The experiment was performed as in (**D**) using the PC, TRAF6 or GAPDH antibodies. (**F**) Confocal immunofluorescence microscopy of PC and MAVS in A549 cells. MAVS/PC and nuclei were stained with FITC/Cy3-conjugated secondary antibodies and DAPI, respectively. (**G**) A549 cells were transfected with HA-TRAF6 for 48 h. HA/PC and nuclei were stained with Cy3/FITC-conjugated secondary antibodies and DAPI, respectively. Scale bars, 10 μm. All experiments were repeated at least three times with consistent results.

Because PC is a mitochondria matrix protein and MAVS is a mitochondria membrane adaptor, the interaction of PC and MAVS requires the translocation of PC from matrix to outer mitochondria. Therefore, we examined whether virus infection triggers PC outer mitochondria translocation. Interestingly, our results showed that virus stimulation caused cytoplasmic translocation of PC after infection for 12 h in A549 cells (Fig. 6A), and similar results were observed in 293T cells (Fig. 6B). Subsequently, the isolation of mitochondrion was performed and mitochondrial extracts were prepared. Co-immunoprecipitation results indicated that the interaction between PC and MAVS was detected in mitochondrial extracts, suggesting that the interaction occurs on mitochondria (Fig. 6C). To further investigate endogenous PC interacted with MAVS-associated components, we performed endogenous co-immunoprecipitation experiments and found that MAVS co-precipitated with PC, showing that they interacted endogenously (Fig. 6D). Similar experiments also revealed that PC was constitutively associated with TRAF6 (Fig. 6E). These results were further supported by confocal microscopy observations that PC and MAVS or TRAF6 co-localized in A549 cells, which suggested that PC had a distribution pattern similar to that of MAVS (Fig. 6F) and TRAF6 (Fig. 6G). These findings demonstrated that PC endogenously interacts with MAVS-associated components.

### Domain mapping of interactions between PC and MAVS or TRAF6

**Figure 7:**
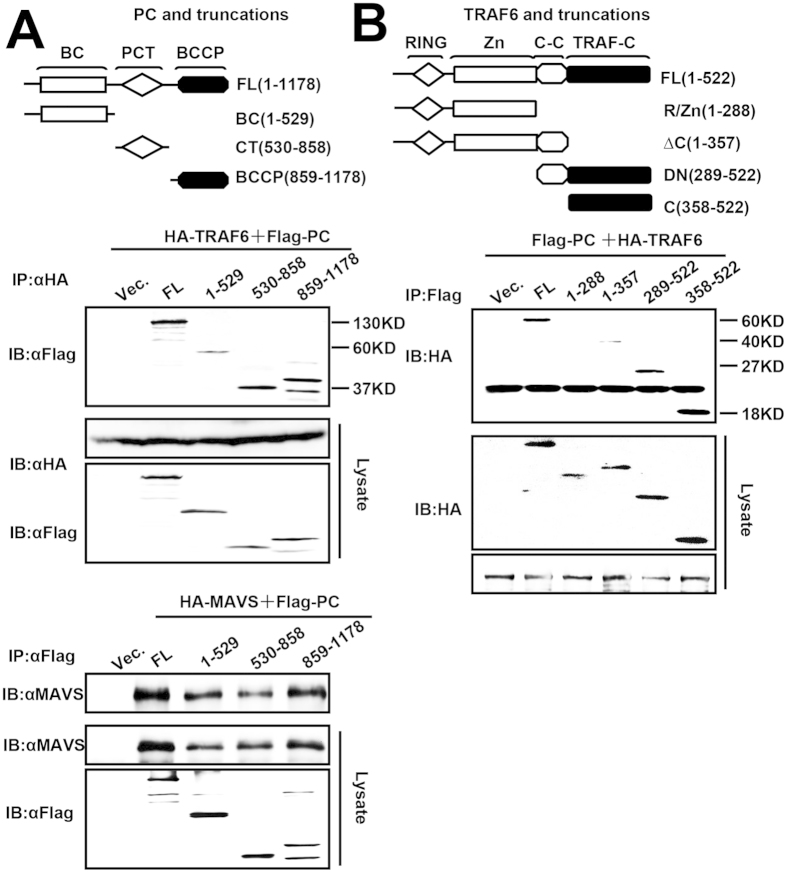
Domain mapping of the interaction between PC and MAVS or TRAF6. (**A**) A schematic presentation of full-length PC (upper panel). HEK293T cells were co-transfected with HA-TRAF6 (middle panel) or HA-MAVS (lower panel) and truncated PC for 48 h. Co-immunoprecipitation and immunoblot analyses were performed with the indicated antibodies. (**B**) A schematic depicting full-length TRAF6 (upper panel). The experiments for the interaction between PC and truncated TRAF6 were performed as described in (**A**) using the indicated antibody (lower panel). All experiments were repeated at least three times with consistent results.

To map the region of PC that interacted with MAVS and TRAF6, we generated a series of Flag-tagged PC truncation mutant constructs (Fig. 7A, upper panel). Surprisingly, all the truncation mutants interacted with TRAF6 (Fig. 7A, middle panel) and MAVS (Fig. 7A, lower panel). Our observation is consistent with a previous report; a similar interaction pattern was identified between MAVS and UBXN1^40^. We next asked which domain of TRAF6 bound to PC and then we generated a series of TRAF6 deletion constructs (Fig. 7B, upper panel). Co-immunoprecipitation experiments showed that PC bound to full-length, aa 289–522, and aa 358–522 fragments, and weakly interacted with aa 1-357 fragment, but failed to bind to aa 1-288 fragment which contained the amino-terminal RING-finger domain and a series of zinc finger domains (Fig. 7B, lower panel). These results indicate that PC predominantly binds to the coiled-coil and C-terminal domain (aa 289–522) of TRAF6.

### PC positively regulates virus-triggered NF-κB signaling

**Figure 8:**
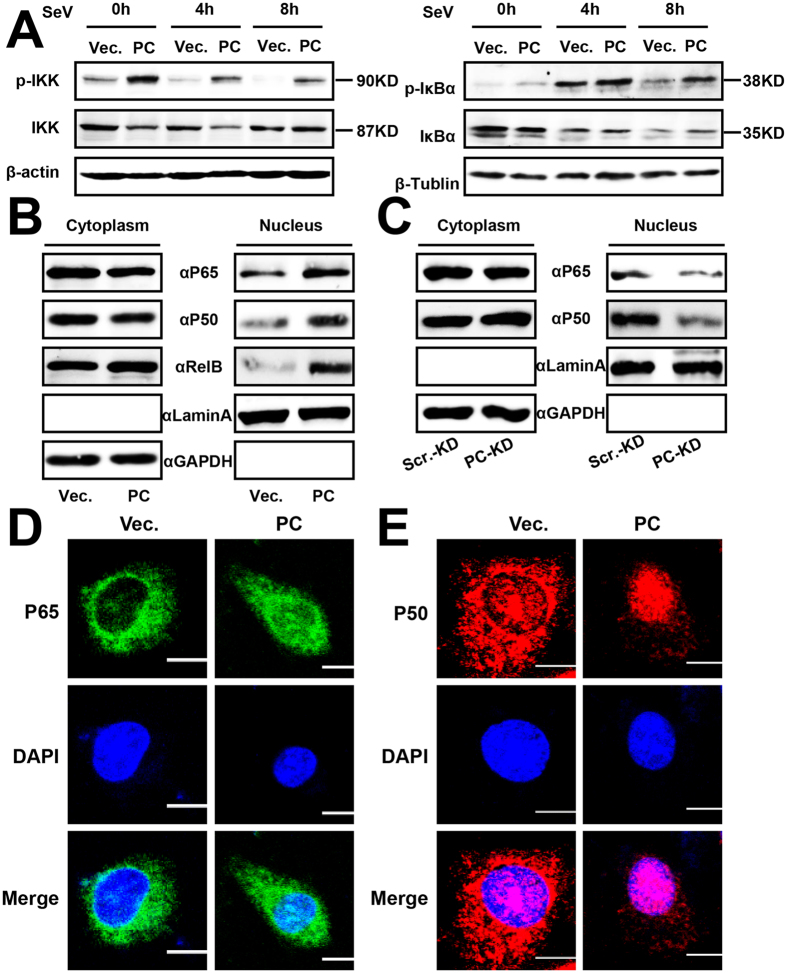
PC positively regulates virus-triggered NF-κB signaling. (**A**) A549 cells were transfected with the PC expression plasmid or vector. Twenty-four hours after transfection, cells were infected with SeV (MOI = 1) for the indicated time points before western blotting. (**B**) A549 cells were transfected with the PC expression plasmid or vector. Twenty-four hours later, cells were infected with SeV (MOI = 1) for 12 h, and cytosolic and nuclear extracts were prepared for western blot. GAPDH and laminA were used as markers for the cytosolic and nuclear fractions, respectively. (**C**) PC-KD and Scr.-KD cells were infected with SeV (MOI = 1) for 12 h. Nuclear extraction experiments were performed as described in (**B**). (**D,E**) A549 cells were transfected with PC expression plasmid or vector for 48 h. After fixation, the cells were immunolabeled with the indicated antibodies. Nuclei were stained with DAPI (blue). Scale bar, 10 μm. All experiments were repeated at least three times with consistent results.

MAVS recruits TRAF6 to activate NF-κB signaling and eventually to induce the expression of IFNs and pro-inflammatory cytokines^1^,^19^. The common step in this activating process is mediated by an IκB kinase (IKK) complex that phosphorylates IκBα and targets it for proteasome degradation, thus promoting NF-κB nuclear translocation^41^,^42^. Since our results showed that PC is involved in virus-triggered induction of IFNs and pro-inflammatory cytokines, and because it targets MAVS-associated components, we next assess the effect of PC on IKK and IκBα phosphorylation. The results showed that PC expression significantly promoted IKK and IκBα phosphorylation (Fig. 8A). Furthermore, we examined the effect of PC on NF-κB translocation from the cytosol to the nucleus. Western blot analyses revealed that P50, P65, and RelB protein levels were significantly increased in the nuclear extracts (Fig. 8B). Stable PC-silenced PC-KD cells were used to investigate the effect of endogenous PC on NF-κB translocation. We found that depletion of PC decreased the NF-κB translocation from the cytoplasm to the nucleus (Fig. 8C). Similar results were observed by immunofluorescence microscopy (Fig. 8D,E). These results are consistent with our observations that PC up-regulates RLR-mediated NF-κB activation and the production of IFNs and pro-inflammatory cytokines.

## Discussion

PC is believed to participate in multiple biological processes, including gluconeogenesis and lipogenesis, neurotransmitter biosynthesis, and glucose-induced insulin secretion by pancreatic islets. After the first discovery of PC in 1959, there has been much progress in understanding the enzyme’s structure and function over the last decades. Although many papers have reported the crystal structure and multifunction of PC, its involvement in the innate immune system has not yet been described. In this study, we identified a previously unrecognized function of PC during viral infection; it exhibits strong antiviral activity toward a broad range of RNA virus infections through its effects on RIG-I-MAVS-NF-κB signaling.

RLR signaling is initiated by the recognition of distinct species of viral dsRNAs by one of the two cytoplasmic sensors: RIG-I or MDA5. Viral RNA binding drives RIG-I and MDA5 to undergo conformational changes that facilitate their recruitment to the mitochondria to interact with the adaptor molecule MAVS, which recruits E3 ligases TRAF3, TRAF6, and other adaptors to form the MAVS signalosome and introduce the production of type I IFNs and pro-inflammatory cytokines^12^,^13^. Though PC was described mainly located in mitochondria, in this study, we discovered that PC could translocate to cytoplasm in response to viral infection, and the interaction between PC and MAVS occurs on mitochondria. These results are consistent with a recent report that demonstrates PC can interact with nonstructural 5A protein of Hepatitis C virus in cytoplasm^43^. Our research indicates that PC induces virus-triggered IFN and inflammatory cytokine expression by targeting MAVS and TRAF6, and specifically inhibiting the replication of RNA viruses. Many studies have shown that mitochondrial proteins facilitate MAVS signaling pathways to inhibit viral replication. ECSIT (evolutionarily conserved signaling intermediate in Toll pathways) localizes to the mitochondria through its N-terminal domain and exerts an antiviral effect by directly interacting with RIG-I, MDA5, and MAVS, and facilitating MAVS recruitment by RIG-I^44^. Translocases of outer membrane 70 (Tom70), a mitochondrial import receptor, interacts with MAVS upon RNA virus infection to enhance IRF3-mediated gene expression and facilitate host antiviral effects^45^. In this study, we demonstrate that PC is a new component of the MAVS signal complex on mitochondria that enhances MAVS-TRAF6 signaling. PC also has strong antiviral effects on RNA viruses such as IAV, EV71, and VSV. However PC interacted with the virus protein NS5A of the HCV and enhanced virion packaging and release without affecting intracellular HCV RNA and protein levels^43^. These results indicate different mechanisms of PC as a mediator of immunity, depending on virus type.

TRAF6 consists of four domains including a RING-finger domain, a domain containing six consecutive zinc fingers, a coiled-coil domain, and a TRAF-C domain^46^. The RING-finger domain confers E3 ligase activity on TRAF6, and the RING and zinc fingers are reportedly critical for the activation of downstream signaling^47^. The coiled-coil and TRAF-C domains facilitate TRAF6 oligomerization and its binding to the upstream receptors or adaptor proteins^46^. Our results show that PC interacts with the coiled-coil and TRAF-C domains of TRAF6, which implies that PC interacts with TRAF6 and regulates its signaling as mentioned above. In contrast, TRAF6 and MAVS interact with all the functional domains of PC. Based on these data, we hypothesize that the PC domain that binds to TRAF6 and MAVS may be of physiological significance. The precise mechanism underlying PC mediation of the interaction between MAVS and TRAF6 needs to be investigated further.

It is well known that NF-κB is present in the cytoplasm in association with inhibitory IκB proteins. Upon viral infection, phosphorylated IKK complexes can phosphorylate IκBα, which is subsequently ubiquitinated and degraded by the proteasome. NF-κB is then released and translocates to the nucleus^12^,^13^. We found that PC increases the phosphorylation levels of IKK and IκBα and promotes NF-κB nuclear translocation. Moreover, PC depletion has the opposite effect. In conclusion, PC enhances the virus-triggered activation of the immune response through RIG-I-MAVS-NF-κB signaling and induces the expression of IFNs and pro-inflammatory cytokines to hamper virus replication and infection.

## Methods

### Cell culture and viruses

A549 cells were cultured in F12K medium containing 10% fetal bovine serum (FBS). 293T, Huh7, and Vero cells were cultured in Dulbecco’s modified Eagle’s medium (DMEM) containing 10% FBS. RD cells were cultured in RPMI 1640 medium containing 10% FBS. All cell cultures were maintained at 37 °C in a 5% CO_2_ incubator. The influenza virus A/Hong Kong/498/97 (H3N2) strain was provided by the China Center for Type Culture Collection. A recombinant VSV carrying the enhanced green fluorescent protein gene (VSV-eGFP) and the EV71 strain were described previously^48^.

### Plasmids and reagents

A human PC full-length cDNA clone was purchased from Proteintech Group. Coding regions for PC or truncated versions were generated by polymerase chain reaction (PCR) amplification using PC cDNA as a template. The PCR products were digested with EcoRI/BamHI and cloned directly into the pCMV-14-3× flag expression vector, digested with HindIII/SalI and cloned into the PKH3-3× HA expression vector, or digested with EcoRI/ HindIII and cloned into pCMV-tag2B expression vector. The overexpression plasmids TRADD, NEMO, TRAF3, MAVS, and TRAF6 were constructed for this study. The coding regions of TRAF6 and TRAF6 truncations were generated by PCR amplification using the TRAF6 expression plasmid as a template. The PCR products were digested with EcoRI/XhoI and cloned directly into the pCAgg-HA expression vector. All primers used for construction are listed in Supplementary Table 1.

The IFN-λ1, IL-6, IL-8, and NF-κB promoter/reporter constructs were also described elsewhere^49^,^50^. The ISRE and IFN-β promoters were provided by Professor Hongbing Shu of Wuhan University, China. All constructs and gift plasmids were confirmed by DNA sequencing. The small interfering RNA (siRNA) plasmids specifically directed against PC and MAVS were purchased from Guangzhou RiboBio Group. The MAVS-specific siRNA sequence was described in previous report^51^.

Antibodies against PC, VP1, TRAF6, MAVS, P65, P50, RelB, and LaminA (Santa Cruz Biotechnology); PKR and MxA (Origene); IKK, p-IKK, IkBα, p-IkBα (Cell Signaling Technology); HA and Flag (MBL); GAPDH and β-tubulin (Invitrogen) were purchased from the indicated manufacturers. PAA (Sigma) and pyruvate carboxylase activity assay kit were purchased from Solarbio Co.

### Measurement of IAV replication

A549 cells were infected with IAV/Hong Kong/498/97 (H3N2) at an MOI of 1. The viral titers were measured at indicated times post-infection with hemagglutination assays in U-shaped plates, as described previously^48^,^52^,^53^. The relative RNA levels of nucleoprotein (NP)-mRNA, NP-cRNA, and NP-viral RNA (vRNA) were detected by qRT-PCR as described previously^48^,^53^. The following primers were used for reverse transcription:

*NP-vRNA*, 5′-CTCACCGAGTGACATCAACATCATG-3′; *NP-cRNA*, 5′-AGTAGAAACAAGGGTATTTTTCTTTAATTGTCAT-3′; and *NP-mRNA*, oligo(dT). The following primers were used for qRT-PCR: *NP*, 5′-ATCAGACCGAACGAGAATCCAGC-3′ (sense) and 5′-GGAGGCCCTCTGTTGATTAGTGT-3′ (antisense)^48^,^52^.

### Production of lentivirus containing specific shRNA

The lentiviral pLKO.1-VRC (TRC) cloning vector was a gift from Professor Yingliang Wu of Wuhan University, China. Lentivirus containing specific shRNA was produced as described in the addgene pLKO.1-VRC cloning vector protocol. Briefly, shRNA against the negative control or PC (shNC or shPC, which was constructed to the pLKO.1-VRC cloning vector), lentivirus packaging plasmids psPAX2, and envelope plasmid pMD2.G were co-transfected into 293T cells in 60-mm culture dishes with Lipofectamine 2000 (Invitrogen). After 24 h, we harvested media, transferred it to a polypropylene storage tube, and then added 5 mL fresh media to the cells and incubated them for another 24 h. The harvested media was spun at 15,000 g for 5 minutes and filtered through a 0.45-μm filter (Millipore Corp) to remove cells. The specific shPC sequence was:

CCGGGCCAAGGAGAACAACGTAGATCTCGAGATCTACGTTGTTCTCCTTGGCTTTTTG. It was obtained from the Sigma website and was described in a previous report^29^.

### Gene silencing with lentivirus encoding specific shRNA

A549 cells were plated in 6-well plates (4 × 10^5^ cells/well). When cells were grown to approximately 70% confluency, they were incubated in fresh culture medium containing 8 μg/mL polybrene. With the addition of control-shRNA or PC-shRNA lentivirus-containing media to the A549 cells, 24 h after infection, virus-infected cells were selected by puromycin (1 μg/mL) for 72 h and assayed.

### Nuclear extraction

The cell samples were collected and washed in cold phosphate-buffered saline (PBS). The cells were resuspended in buffer A (10 mM Tris-HCl [pH 7.4], 5 mM MgCl_2_, 10 mM NaCl, 1 mM DTT, 10% protease inhibitor mixture) for 15 min on ice before 0.5% NP-40 in buffer A was added, and the mixture was vortexed for 10 s. Nuclei were pelleted by centrifugation at 15,000 g for 1 min, and cytosolic protein-containing supernatants were collected. After the pellet was washed in buffer A, it was resuspended in buffer C (20 mM HEPES-KOH [pH 7.9], 1.5 mM MgCl_2_, 0.5 M NaCl, 1 mM DTT, 0.2 mM EDTA, 1% NP-40, 10% protease mixture inhibitor), vortexed for 15 s, and incubated on ice for 10 min; this process was repeated three times. After centrifugation at 15,000 g for 30 min, nuclei protein-containing supernatants were collected. The indicated protein was detected by western blot analysis.

### VSV plaque assays

The target cells were grown in 12-well plates and transfected with the indicated plasmids. Twenty-four hours after transfection, cells were infected with VSV (MOI = 1). After 1 h, cells were washed with warm PBS, and fresh medium was added. After 24 h, the supernatants were harvested, diluted to 10^−6^, 10^−5^, 10^−4^, 10^−3^, 10^−2^, and 10^−1^, and used to infect confluent Vero cells cultured on 24-well plates. At 1 h post-infection, supernatants were removed, and a mixture of warm 3% low melting-point agarose and fresh medium were overlaid. At 72 h post-infection, cells were stained with 0.2% crystal violet for 2 h, and then the overlay was removed. Plaques were counted, averaged, and multiplied by the dilution factor to determine the viral titer (PFU/mL).

### Confocal immunofluorescence

The target cells were fixed with equal volumes of methyl alcohol and acetone for 15 min, washed three times with PBS, and blocked with PBS containing 4% bovine serum albumin (BSA) for 1 h at room temperature. Then, the cells were incubated with the primary antibody overnight at 4 °C and washed three times with PBS containing 0.01% Tween 20 and 1% BSA. After incubation with the secondary antibodies (ProteinTech Group) for 1 h, the samples were washed five times with PBS containing 0.01% Tween 20 and 1% BSA. Mounting was performed with Vectashield mounting medium with DAPI (Vector Laboratories), and the cells were visualized by confocal laser microscopy (FLUOVIEW FV1000; Olympus, Tokyo, Japan).

### MTS Cell Viability Assay

A549 cells were seeded into 96-well plates at 5000 cells/well. After cell adhesion, the PC-specific siRNA or control RNA were then transfected into A549 cells using Lipofectamine 2000 reagent (Invitrogen). Forty-eight hours after transfection, MTS assays were performed using the MTS cell viability kit (Promega) according to the manufacturer’s instructions. Cell viability was then calculated.

### Isolation of mitochondria

Cells were collected by centrifugation, then suspended in buffer A (250 mM Sucrose, 10 mM Tris-HCl pH7.4, 1 mM EDTA), homogenized in a pre-chilled Dounce homogenizer (Kontes), and postnuclear supernatant collected. Mitochondrion were sedimented at 13,000 g for 10 min, washed once in the buffer A, and further purified by centrifugation at 40,000 g for 1 h at 4 °C on a sucrose gradient (17, 31, 42, 50%) in TE buffer (10 mM Tris-HCl pH 7.4, 20 mM EDTA). Mitochondrion were harvested from the 42% and 50%M sucrose interface^54^.

Real-time PCR (qPCR) analysis, Transfection and luciferase reporter assays and Western blot analysis and co-immunoprecipitation can be found in supplementary information and Supplementary Table 2.

### Statistical Analysis

All experiments were reproducible and were carried out in triplicate or quadruplicate. Each set of experiments was repeated at least three times with similar results, and a representative one is shown. The results are presented at the means ± s.d. Student’s t test for paired samples was used to determine statistical significance. Differences were considered statistically significant at a value of *P* < 0.05 was considered statistically significant.

## Additional Information

**How to cite this article**: Cao, Z. *et al.* Pyruvate Carboxylase Activates the RIG-I-like Receptor-Mediated Antiviral Immune Response by Targeting the MAVS signalosome. *Sci. Rep.* **6**, 22002; doi: 10.1038/srep22002 (2016).

## Supplementary Material

Supplementary Information

## References

[b1] Akira S., Uematsu S. & Takeuchi O. Pathogen recognition and innate immunity. Cell 124, 783–801, doi: 10.1016/j.cell.2006.02.015 (2006). 16497588

[b2] Hiscott J. Convergence of the NF-kappaB and IRF pathways in the regulation of the innate antiviral response. Cytokine Growth Factor Rev 18, 483–490, doi: 10.1016/j.cytogfr.2007.06.002 (2007). 17706453

[b3] Kawai T. *et al.* IPS-1, an adaptor triggering RIG-I- and Mda5-mediated type I interferon induction. Nat Immunol 6, 981–988, doi: 10.1038/ni1243 (2005). 16127453

[b4] Takeda K. & Akira S. Toll-like receptors in innate immunity. Int Immunol 17, 1–14, doi: 10.1093/intimm/dxh186 (2005). 15585605

[b5] Kawai T. & Akira S. Toll-like receptors and their crosstalk with other innate receptors in infection and immunity. Immunity 34, 637–650, doi: 10.1016/j.immuni.2011.05.006 (2011). 21616434

[b6] Loo Y. M. & Gale M. Jr. Immune signaling by RIG-I-like receptors. Immunity 34, 680–692, doi: 10.1016/j.immuni.2011.05.003 (2011). 21616437 PMC3177755

[b7] Yoneyama M. *et al.* The RNA helicase RIG-I has an essential function in double-stranded RNA-induced innate antiviral responses. Nat Immunol 5, 730–737, doi: 10.1038/ni1087 (2004). 15208624

[b8] Saito T. *et al.* Regulation of innate antiviral defenses through a shared repressor domain in RIG-I and LGP2. Proc Natl Acad Sci USA 104, 582–587, doi: 10.1073/pnas.0606699104 (2007). 17190814 PMC1766428

[b9] Yoneyama M. *et al.* Shared and unique functions of the DExD/H-box helicases RIG-I, MDA5, and LGP2 in antiviral innate immunity. J Immunol 175, 2851–2858 (2005). 16116171 10.4049/jimmunol.175.5.2851

[b10] Jacobs J. L. & Coyne C. B. Mechanisms of MAVS regulation at the mitochondrial membrane. J Mol Biol 425, 5009–5019, doi: 10.1016/j.jmb.2013.10.007 (2013). 24120683 PMC4562275

[b11] Meylan E. *et al.* Cardif is an adaptor protein in the RIG-I antiviral pathway and is targeted by hepatitis C virus. Nature 437, 1167–1172, doi: 10.1038/nature04193 (2005). 16177806

[b12] Seth R. B., Sun L., Ea C. K. & Chen Z. J. Identification and characterization of MAVS, a mitochondrial antiviral signaling protein that activates NF-kappaB and IRF 3. Cell 122, 669–682, doi: 10.1016/j.cell.2005.08.012 (2005). 16125763

[b13] Xu L. G. *et al.* VISA is an adapter protein required for virus-triggered IFN-beta signaling. Mol Cell 19, 727–740, doi: 10.1016/j.molcel.2005.08.014 (2005). 16153868

[b14] Yoneyama M., Onomoto K., Jogi M., Akaboshi T. & Fujita T. Viral RNA detection by RIG-I-like receptors. Curr Opin Immunol 32, 48–53, doi: 10.1016/j.coi.2014.12.012 (2015). 25594890

[b15] Guo B. & Cheng G. Modulation of the interferon antiviral response by the TBK1/IKKi adaptor protein TANK. J Biol Chem 282, 11817–11826, doi: 10.1074/jbc.M700017200 (2007). 17327220

[b16] Fitzgerald K. A. *et al.* IKKepsilon and TBK1 are essential components of the IRF3 signaling pathway. Nat Immunol 4, 491–496, doi: 10.1038/ni921 (2003). 12692549

[b17] Michallet M. C. *et al.* TRADD protein is an essential component of the RIG-like helicase antiviral pathway. Immunity 28, 651–661, doi: 10.1016/j.immuni.2008.03.013 (2008). 18439848

[b18] Hornung V. *et al.* 5′-Triphosphate RNA is the ligand for RIG-I. Science 314, 994–997, doi: 10.1126/science.1132505 (2006). 17038590

[b19] Nakhaei P., Genin P., Civas A. & Hiscott J. RIG-I-like receptors: sensing and responding to RNA virus infection. Semin Immunol 21, 215–222, doi: 10.1016/j.smim.2009.05.001 (2009). 19539500

[b20] Liu S. *et al.* Phosphorylation of innate immune adaptor proteins MAVS, STING, and TRIF induces IRF3 activation. Science 347, aaa2630, doi: 10.1126/science.aaa2630 (2015). 25636800

[b21] Xia P. *et al.* IRTKS negatively regulates antiviral immunity through PCBP2 sumoylation-mediated MAVS degradation. Nat Commun 6, 8132, doi: 10.1038/ncomms9132 (2015). 26348439 PMC4569712

[b22] Durbin J. E. *et al.* Type I IFN modulates innate and specific antiviral immunity. J Immunol 164, 4220–4228 (2000). 10754318 10.4049/jimmunol.164.8.4220

[b23] Levy D. E. & Garcia-Sastre A. The virus battles: IFN induction of the antiviral state and mechanisms of viral evasion. Cytokine Growth Factor Rev 12, 143–156 (2001). 11325598 10.1016/s1359-6101(00)00027-7

[b24] Levy D. E. & Marie I. J. RIGging an antiviral defense–it’s in the CARDs. Nat Immunol 5, 699–701, doi: 10.1038/ni0704-699 (2004). 15224097

[b25] Lietzan A. D. & St Maurice M. Functionally diverse biotin-dependent enzymes with oxaloacetate decarboxylase activity. Arch Biochem Biophys 544, 75–86, doi: 10.1016/j.abb.2013.10.014 (2014). 24184447

[b26] Jitrapakdee S. *et al.* Structure, mechanism and regulation of pyruvate carboxylase. Biochem J 413, 369–387, doi: 10.1042/BJ20080709 (2008). 18613815 PMC2859305

[b27] Jitrapakdee S., Vidal-Puig A. & Wallace J. C. Anaplerotic roles of pyruvate carboxylase in mammalian tissues. Cell Mol Life Sci 63, 843–854, doi: 10.1007/s00018-005-5410-y (2006). 16505973 PMC11136034

[b28] Owen O. E., Kalhan S. C. & Hanson R. W. The key role of anaplerosis and cataplerosis for citric acid cycle function. J Biol Chem 277, 30409–30412, doi: 10.1074/jbc.R200006200 (2002). 12087111

[b29] Sellers K. *et al.* Pyruvate carboxylase is critical for non-small-cell lung cancer proliferation. J Clin Invest 125, 687–698, doi: 10.1172/JCI72873 (2015). 25607840 PMC4319441

[b30] Thonpho A., Rojvirat P., Jitrapakdee S. & MacDonald M. J. Characterization of the distal promoter of the human pyruvate carboxylase gene in pancreatic beta cells. PLoS One 8, e55139, doi: 10.1371/journal.pone.0055139 (2013). 23383084 PMC3559343

[b31] Robinson B. H. Lactic acidemia and mitochondrial disease. Mol Genet Metab 89, 3–13, doi: 10.1016/j.ymgme.2006.05.015 (2006). 16854608

[b32] Huang X. *et al.* Heterotrimerization of the growth factor receptors erbB2, erbB3, and insulin-like growth factor-i receptor in breast cancer cells resistant to herceptin. Cancer Res 70, 1204–1214, doi: 10.1158/0008-5472.CAN-09-3321 (2010). 20103628

[b33] Lee J. H. *et al.* Toxicity generated through inhibition of pyruvate carboxylase and carnitine palmitoyl transferase-1 is similar to high glucose/palmitate-induced glucolipotoxicity in INS-1 beta cells. Mol Cell Endocrinol 383, 48–59, doi: 10.1016/j.mce.2013.12.002 (2014). 24333689

[b34] Park S. S., Kim S. J., Choi H., Chang C. & Kim E. TR4 promotes fatty acid synthesis in 3T3-L1 adipocytes by activation of pyruvate carboxylase expression. FEBS Lett 588, 3947–3953, doi: 10.1016/j.febslet.2014.09.007 (2014). 25240193

[b35] Zeczycki T. N., Maurice M. S. & Attwood P. V. Inhibitors of Pyruvate Carboxylase. Open Enzym Inhib J 3, 8–26, doi: 10.2174/1874940201003010008 (2010). 22180764 PMC3238542

[b36] Pauli E. K. *et al.* Influenza A virus inhibits type I IFN signaling via NF-kappaB-dependent induction of SOCS-3 expression. PLoS Pathog 4, e1000196, doi: 10.1371/journal.ppat.1000196 (2008). 18989459 PMC2572141

[b37] Prescott J. *et al.* New World hantaviruses activate IFNlambda production in type I IFN-deficient vero E6 cells. PLoS One 5, e11159, doi: 10.1371/journal.pone.0011159 (2010). 20567522 PMC2887373

[b38] Kell A. M. & Gale M. Jr. RIG-I in RNA virus recognition. Virology 479–480C, 110–121, doi: 10.1016/j.virol.2015.02.017 (2015). PMC4424084 25749629

[b39] Loo Y. M. *et al.* Distinct RIG-I and MDA5 signaling by RNA viruses in innate immunity. J Virol 82, 335–345, doi: 10.1128/JVI.01080-07 (2008). 17942531 PMC2224404

[b40] Wang P. *et al.* UBXN1 interferes with Rig-I-like receptor-mediated antiviral immune response by targeting MAVS. Cell Rep 3, 1057–1070, doi: 10.1016/j.celrep.2013.02.027 (2013). 23545497 PMC3707122

[b41] Scheidereit C. IkappaB kinase complexes: gateways to NF-kappaB activation and transcription. Oncogene 25, 6685–6705, doi: 10.1038/sj.onc.1209934 (2006). 17072322

[b42] Hayden M. S., West A. P. & Ghosh S. NF-kappaB and the immune response. Oncogene 25, 6758–6780, doi: 10.1038/sj.onc.1209943 (2006). 17072327

[b43] Yim S. A., Lim Y. S., Kim J. W. & Hwang S. B. Nonstructural 5A protein of hepatitis C virus interacts with pyruvate carboxylase and modulates viral propagation. PLoS One 8, e68170, doi: 10.1371/journal.pone.0068170 (2013). 23861867 PMC3701667

[b44] Lei C. Q. *et al.* ECSIT bridges RIG-I-like receptors to VISA in signaling events of innate antiviral responses. J Innate Immun 7, 153–164, doi: 10.1159/000365971 (2015). 25228397 PMC6738808

[b45] Liu X. Y., Wei B., Shi H. X., Shan Y. F. & Wang C. Tom70 mediates activation of interferon regulatory factor 3 on mitochondria. Cell Res 20, 994–1011, doi: 10.1038/cr.2010.103 (2010). 20628368

[b46] Inoue J., Gohda J. & Akiyama T. Characteristics and biological functions of TRAF6. Adv Exp Med Biol 597, 72–79 (2007). 17633018 10.1007/978-0-387-70630-6_6

[b47] Yamazaki K. *et al.* Two mechanistically and temporally distinct NF-kappaB activation pathways in IL-1 signaling. Sci Signal 2, ra66, doi: 10.1126/scisignal.2000387 (2009). 19843958

[b48] Wang Q. *et al.* Soluble interleukin-6 receptor-mediated innate immune response to DNA and RNA viruses. J Virol 87, 11244–11254, doi: 10.1128/JVI.01248-13 (2013). 23946454 PMC3807281

[b49] Yu Y. *et al.* Hepatitis B virus induces a novel inflammation network involving three inflammatory factors, IL-29, IL-8, and cyclooxygenase-2. J Immunol 187, 4844–4860, doi: 10.4049/jimmunol.1100998 (2011). 21957142

[b50] Liu S. *et al.* Major vault protein: a virus-induced host factor against viral replication through the induction of type-I interferon. Hepatology 56, 57–66, doi: 10.1002/hep.25642 (2012). 22318991

[b51] Matsushima-Miyagi T. *et al.* TRAIL and Noxa are selectively upregulated in prostate cancer cells downstream of the RIG-I/MAVS signaling pathway by nonreplicating Sendai virus particles. Clin Cancer Res 18, 6271–6283, doi: 10.1158/1078-0432.CCR-12-1595 (2012). 23014529

[b52] Li W. *et al.* Activation of interleukin-32 pro-inflammatory pathway in response to influenza A virus infection. PLoS One 3, e1985, doi: 10.1371/journal.pone.0001985 (2008). 18414668 PMC2288676

[b53] Mukhtar M. M. *et al.* Single-chain intracellular antibodies inhibit influenza virus replication by disrupting interaction of proteins involved in viral replication and transcription. Int J Biochem Cell Biol 41, 554–560, doi: 10.1016/j.biocel.2008.07.001 (2009). 18687409

[b54] Zhang X. *et al.* MicroRNA directly enhances mitochondrial translation during muscle differentiation. Cell 158, 607–619, doi: 10.1016/j.cell.2014.05.047 (2014). 25083871 PMC4119298

