# Supplementary material for: Pyruvate Carboxylase Activates the RIG-I-like Receptor-Mediated Antiviral Immune Response by Targeting the MAVS signalosome
Source: Sci Rep. 2016 Feb 24;6:22002. doi: 10.1038/srep22002 (PMC4764940; doi:10.1038/srep22002)
Supplement: Supplementary Information [file srep22002-s1.pdf]

Pyruvate Carboxylase Activates the RIG-I-like Receptor-Mediated Antiviral Immune Response by Targeting the MAVS signalosome

Zhongying Cao; Yaqin Zhou; Shengli Zhu; Jian Feng; Xueyuan Chen; Shi Liu; Nanfang Peng; Xiaodan Yang; Gang Xu; Ying Zhu*

Supplementary Table 1 : sequence primers of construction in this paper.

gene	5’primer (5’ to 3’)	3’primer (5’ to 3’)
3×Flag-PC	GCTGAATTCGCCACCATGC TGAAGTTCCGAACAGT	TCAGGATCCCTCGATCTCCA GGATGAGGTCGTCA
3×Flag-PC-BC (1-529 aa)	ACGGAATTCGCCACCATGC TGAAGTTCCGAACAG	TATGGATCCGACAACGGGGT CCGTGGGGC
3×Flag-PC-PCT (530-858 aa)	TAGGAATTCGCCACCATGC CTGCAGTGCCCATAGGCC	CGCGGATCCGTTGCCAGACT TCATGGTGGCCGT
3×Flag-PC-BCCP(859-1178 aa)	GCGGAATTCGCCACCATGT CGGACGTGTATGAAAATG	CGCGGATCCCTCGATCTCCA GGATGAGGTCGTC
HA-TRAF6	GCGGAATTCGCCACCATGA GTCTGCTAAACTGTGA	TCGCTCGAGTACCCCTGCAT CAGTACTTCGTGGCT
HA-TRAF6-R/Zn	GCGGAATTCGCCACCATGA	GCGCTCGAGATACCCAGAGT

(1-288 aa)	GTCTGCTAAACTGTGA	CGGGTATAACGCTCA
HA-TRAF6-ΔC(1-357)	GCGGAATTCGCCACCATGAGTCTGCTAAACTGTGA	CGGCTCGAGAATCTTCCAAATATAAATTCCATTGCACTGC
HA-TRAF6-DN (289-522 aa)	TCTGAATTCGCCACCATGATCTCAGAGGTCCGGAA	TCGCTCGAGTACCCCTGCATCAGTACTTCGTGGCT
HA-TRAF6-C (358-522 aa)	GCGGAATTCGCCACCATGAACTTTGGAATGCATTT	TCGCTCGAGTACCCCTGCATCAGTACTTCGTGGCT

Real-time PCR (qPCR) analysis

Quantitative RT-PCR analysis was performed to determine relative mRNA levels. Total RNA was isolated with TRIzol (Invitrogen). Cellular RNA samples were reverse-transcribed with oligo(dT) primers. qPCR was performed in a Light Cycler 480 (Roche, Basel, Switzerland). GAPDH was amplified as an internal control, and the used primers are listed below:

Supplementary Table 2: sequence primers of qPCR in this paper.

gene	5'primer (5' to 3')	3'primer (5' to 3')
PC	CCTTTGGGAATGGGGCGCTGTTTGT	ACAGAGTCGCTGGTGAGCCGAGTCC
IFN-α	TTTCTCCTGCCTGAAGGACAG	GCTCATGATTTCTGCTCTGACA
IFN-β	AAAGAAGCAGCAATTTTCAGC	CCTTGGCCTTCAGGTAATGCA
IFN-λ1	CTTCCAAGCCCACCCCAACT	GGCCTCCAGGACCTTCAGC
Mx1	GCCGGCTGTGGATATGCTA	TTTATCGAAACATCTGTGAAAGCAA

PKR	AGAGTAACCGTTGGTGACATAACCT	GCAGCCTCTGCAGCTCTATGTT
IL-6	GGTACATCCTCGACGGCATCTCA	TGCACAGCTCTGGCTTGTTCCTC
IL-8	GGTGCAGTTTTGCCAAGGAG	TTCCTTGGGGTCCAGACAGA
IL-1β	CAGAAGTACCTGAGCTCGCC	CATGGCCACAACAACTGACG
TNFα	CTTCTCGAACCCCGAGTGAC	ATGAGGTACAGGCCCTCTGA

Transfection and luciferase reporter assays

Cells were plated in 6-well plates (4×10^5 cells/well) or 24-well plates (1×10^5 cells/well) and grown to 80% confluence before they were transfected with the indicated overexpression plasmids, reporter plasmids and a *Renilla* luciferase reporter vector pRL-TK were cotransfected using Lipofectamine 2000 (Invitrogen). Twenty-four hours after transfection, cells were infected with Sendai virus (SeV) for 12 h and then harvested. The Dual-Luciferase reporter assay system (Promega) was used to measure the luciferase activity of each sample. *Renilla* luciferase activities were determined as internal controls to confirm transfection efficiency.

Western blot analysis and co-immunoprecipitation

Whole cell lysates were prepared by suspending cells in lysis buffer (0.01% EDTA, 0.1% Triton X-100, and 10% proteinase inhibitor mixture), sonicating, and centrifuging at 15,000 g for 15 min (Li et al., 2008). The supernatants were pre-cleared by incubating with protein G PLUS-Agarose beads (Roche) for 1 h and

then centrifuged at 15,000 g for 1 min. The supernatants were incubated with the indicated antibody and cross-linked to protein G PLUS-Agarose beads. Beads were washed five times before the proteins were eluted by boiling for 10 min in SDS sample lysis buffer. Immunoblots were visualized with an Enhance Chemiluminescent Detection System (Pierce).

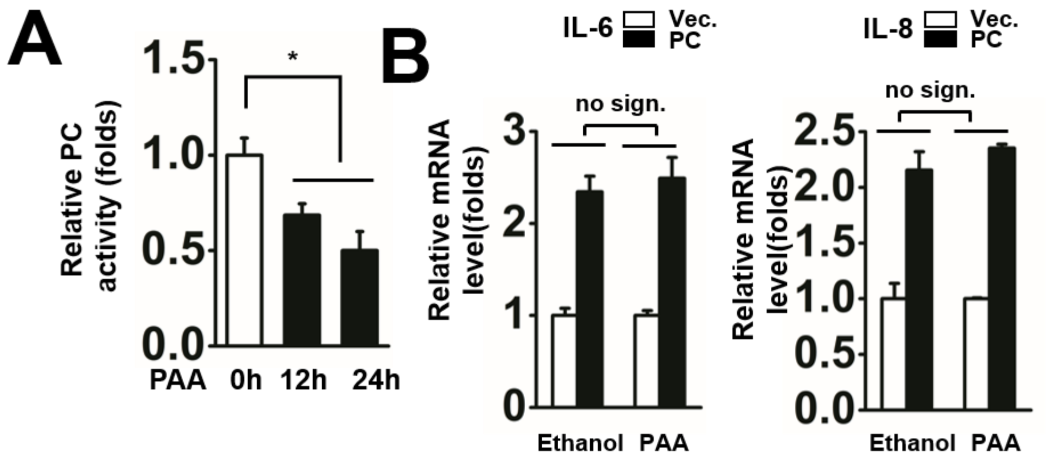

Supplemental Figure 1: (A) A549 cells were incubated with 2.5 mM phenylacetic acid (PAA) for the indicated times, and PC activity assays were performed according to the manufacture protocol. (B) A549 cells were transfected with vector or PC expression plasmids for 24 h and then treated with 2.5 mM phenylacetic acid (PAA) or with ethanol control for 24 h. The mRNA level of IFN-β and IFN-λ1 were detected by qPCR after SeV (MOI = 1) infected for 6 h. *$p < 0.05$, no sign. no significant difference (one-way ANOVA).
